# *Mgat5* Deficiency in T Cells and Experimental Autoimmune Encephalomyelitis

**DOI:** 10.5402/2011/374314

**Published:** 2011-08-17

**Authors:** Ani Grigorian, Michael Demetriou

**Affiliations:** ^1^Department of Neurology, University of California, Irvine, CA 92868-4280, USA; ^2^Institute for Immunology, University of California, Irvine, CA 92697-4120, USA; ^3^Department of Microbiology and Molecular Genetics, University of California, Irvine, CA 92697-4025, USA

## Abstract

Multiple sclerosis (MS) is an inflammatory demyelinating and neurodegenerative disease initiated by autoreactive T cells. *Mgat*5, a gene in the Asn (N-) linked protein glycosylation pathway, associates with MS severity and negatively regulates experimental autoimmune encephalomyelitis (EAE) and spontaneous inflammatory demyelination in mice. N-glycan branching by *Mgat*5 regulates interaction of surface glycoproteins with galectins, forming a molecular lattice that differentially controls the concentration of surface glycoproteins. T-cell receptor signaling, T-cell proliferation, T_H_1 differentiation, and CTLA-4 endocytosis are inhibited by *Mgat*5 branching. Non-T cells also contribute to MS pathogenesis and express abundant *Mgat*5 branched N-glycans. Here we explore whether *Mgat*5 deficiency in myelin-reactive T cells is sufficient to promote demyelinating disease. Adoptive transfer of myelin-reactive Mgat5^−/−^ T cells into Mgat5^+/+^ versus Mgat5^−/−^ recipients revealed more severe EAE in the latter, suggesting that *Mgat*5 branching deficiency in recipient naive T cells and/or non-T cells contribute to disease pathogenesis.

## 1. Introduction

**Figure 1:**
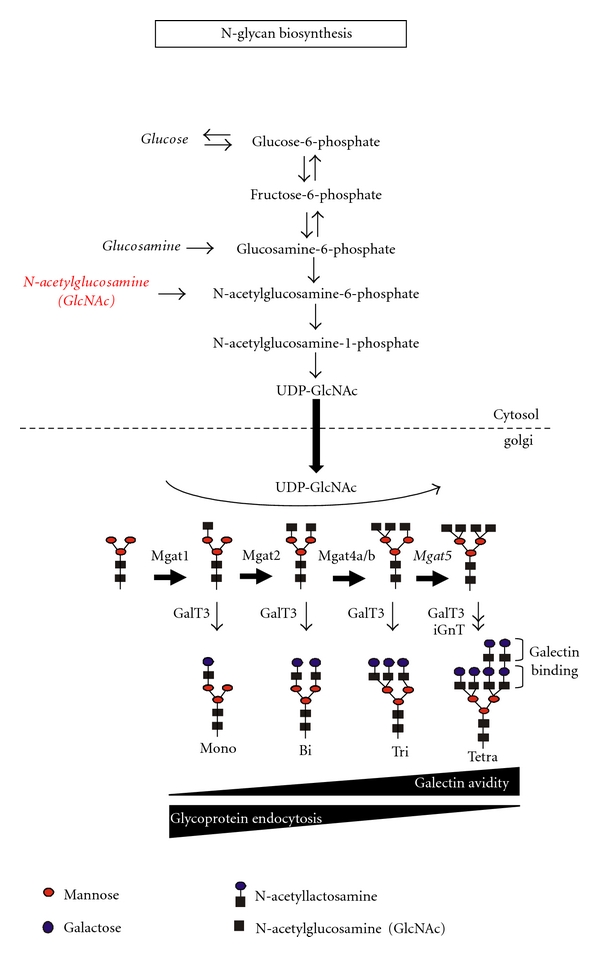
GlcNAc-branched N-glycan biosynthesis. UDP-GlcNAc is required by the N-acetylglucosaminyltransferases Mgat1, 2, 4, and 5 and iGnT. Cytosolic UDP-GlcNAc enters the Golgi via antiporter exchange with Golgi UMP, a reaction product of the N-acetylglucosaminyltransferases. Galectins bind N-acetyllactosamine, with avidity increasing in proportion to the number of N-acetyllactosamine units (i.e., branching). *β*-1,6-GlcNAc-branching by *Mgat*5 promotes poly-N-acetyllactosamine production, further enhancing avidity for galectins. GalT3, galactosyltransferase 3.

Multiple sclerosis (MS) is a complex trait disease where multiple genetic and environmental factors combine to influence susceptibility to disease [1]. Concordance rates in monozygotic twins is ~30%, an ~300-fold higher risk than the general population risk of ~0.1% [2]. However, Baranzini et al. recently reported that they failed to observe sequence differences in the genome, epigenome, or transcriptome of monozygotic twins discordant for MS [3], implicating direct environmental impact on genetic risk. The disparate prevalence of MS along north-south gradients implicates various environmental factors as well, including sunshine exposure, diet, and Vitamin D_3_ status [4]. Genomewide association studies (GWAS) and candidate gene investigations have identified a number of genes associated with MS susceptibility [5–9]. A recent GWAS for variants regulating MS severity identified *Mgat*5, a gene encoding an enzyme in the Asn (N-) linked protein glycosylation pathway [10]. *Mgat*5 catalyzes the addition of *β*1,6-GlcNAc to N-glycan intermediates on glycoproteins in the Golgi apparatus (Figure 1) [11, 12]. Indeed, we have recently shown that multiple genetic and environmental risk factors converge to dysregulate N-glycosylation in MS [13]. N-glycan branching by *Mgat*5 and related enzymes determines binding avidity of surface glycoproteins for galectins, interactions that form a molecular lattice at the cell surface [14]. The galectin-glycoprotein lattice regulates cell growth and differentiation by altering the concentration of surface glycoproteins [15]. Mice deficient in *Mgat*5 display enhanced delayed-type hypersensitivity, spontaneous kidney autoimmunity, and increased susceptibility to experimental autoimmune encephalomyelitis (EAE) [16]. Furthermore, mouse strains susceptible to EAE (PL/J, SJL, and NOD) display reduced N-glycan branching in T cells compared with strains resistant to EAE (129/Sv, BALB/c, and B10.S) [17]. The PL/J strain displays the lowest levels of N-glycan branching with mass spectroscopy and enzyme assays demonstrating deficiencies in multiple N-glycosylation pathway enzymes. A small minority of aged PL/J mice develop a spontaneous late-onset clinical disease manifested by inflammatory demyelination and neurodegeneration, phenotypes markedly enhanced by *Mgat*5^+/−^ and *Mgat*5^−/−^ genotypes in a gene dose-dependent manner. *Mgat*5^−/−^ PL/J mice with spontaneous disease display features of chronic MS, including slow progressive paralysis, tremor, focal dystonic posturing, paroxysmal dystonia, neuronophagia, and axonal damage in demyelinated lesions and normal white matter [17–19]. Increasing N-glycan branching in T cells by metabolically increasing availability of substrate (i.e., UDP-GlcNAc) to *Mgat*5 in the Golgi suppresses autoimmune pathogenesis. *In vitro *supplementation of encephalitogenic T cells with the simple sugar N-acetylglucosamine (GlcNAc), which enhances metabolic supply of UDP-GlcNAc to *Mgat*5, reduced the incidence and severity of EAE following adoptive transfer of the cells into naïve recipient mice [20]. Oral GlcNAc also reduced the development of spontaneous diabetes in nonobese diabetic mice [20].

In T cells, *Mgat*5 branching and the galectin lattice inhibit basal and activation signaling, through the T-cell receptor (TCR) and CD45 in resting cells, promote growth arrest by cytotoxic T-lymphocyte antigen-4 (CTLA-4) in blasting cells and enhance T_H_2 over T_H_1/ T_H_17 differentiation [15, 16, 21–26]. These T-cell-specific phenotypes are consistent with enhanced susceptibility to demyelinating disease in *Mgat*5-deficient mice; however, they do not exclude disease promotion by non-T cells. Here we investigate whether *Mgat*5 branching deficiency in myelin-reactive T cells is sufficient to promote EAE. 

## 2. Materials and Methods

### 2.1. Experimental Autoimmune Encephalomyelitis (EAE) Induction

Adoptive transfer EAE was induced by subcutaneous immunization of *Mgat*5^−/−^ PL/J mice with 100 *μ*g of bovine myelin basic protein (MBP) (Sigma) emulsified in complete Freund's adjuvant containing 4 mg/mL heat-inactivated *Mycobacterium tuberculosis* (H37RA; Difco) distributed over two spots on the hind flank. Splenocytes were harvested 10 days following immunization and stimulated *in vitro* with 50 *μ*g/mL MBP. After 48 h of incubation, CD3^+^ T cells were purified by negative selection (R&D Systems). 2.7 million CD3^+^ T cells were injected intraperitoneally into naïve PL/J *Mgat*5^+/+^ (*n* = 7) and *Mgat*5^−/−^ (*n* = 8) mice. Trypan blue exclusion determined <5% dead cells prior to injection. Mice were weighed and examined daily for clinical signs of EAE over the next 40 days with the observer blinded to experimental conditions. Mice were scored daily in a blinded fashion as follows: 0, no disease; 1, loss of tail tone; 2, hindlimb weakness; 3, hindlimb paralysis; 4, forelimb weakness or paralysis and hindlimb paralysis; 5, moribund or dead. All procedures and protocols with mice were approved by the Institutional Animal Care and Use Committee of the University of California, Irvine, Calif, USA.

### 2.2. Cytokine Measurement

Supernatant from splenocyte cultures simulated with 50 *μ*g/mL bovine MBP (Sigma) for 48 hours were tested for IFN-*γ* and TNF-*α* levels by a multiplexing immunoassay, a bead-based analyte detection system using flow cytometry (FlowCytomix; eBioscience). 

### 2.3. Statistical Analysis

Statistical analysis and *P* values for EAE disease incidence was determined by Fisher's exact test. *P* values for EAE mean clinical score, disease duration, and the highest clinical score were determined by the Mann-Whitney test.

## 3. Results

**Figure 2:**
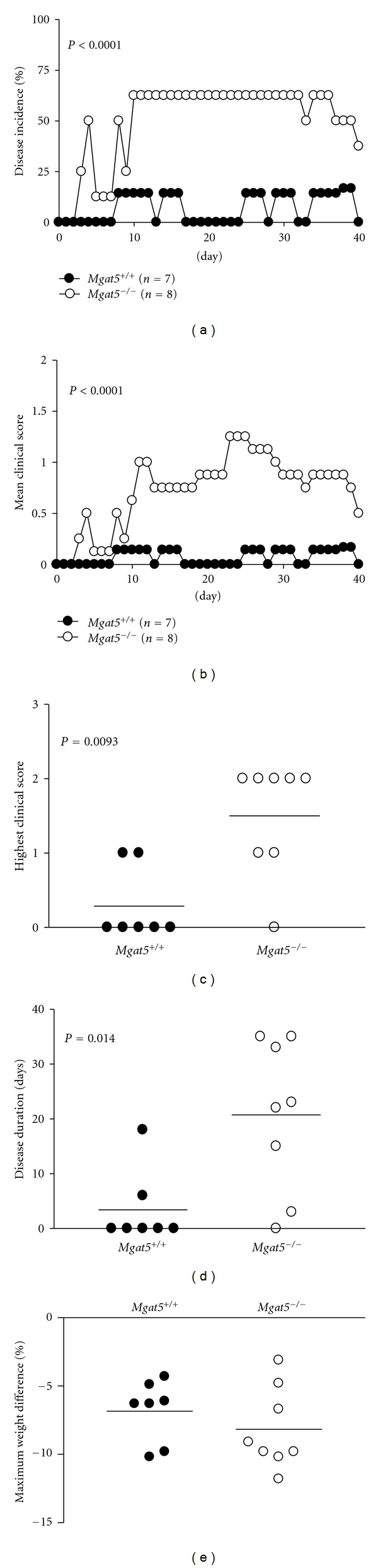
Mice deficient in *Mgat*5 are more susceptible to EAE. (a–e), Splenocytes were isolated from *Mgat*5^−/−^ mice 10 days after immunization with MBP + complete Freund's adjuvant (CFA) and restimulated *in vitro* with MBP for two days. 2.7 million CD3^+^ T cells were injected into naïve *Mgat*5^+/+^ or *Mgat*5^−/−^ mice and scored for clinical signs of EAE daily for 40 days. Mice were weighed daily throughout the duration of the experiment and used to determine maximum weight fluctuations. P value for EAE incidence was determined by Fisher's exact test. *P* values for EAE mean clinical score, disease duration, and the highest clinical score were determined by the Mann-Whitney test.

**Figure 3:**
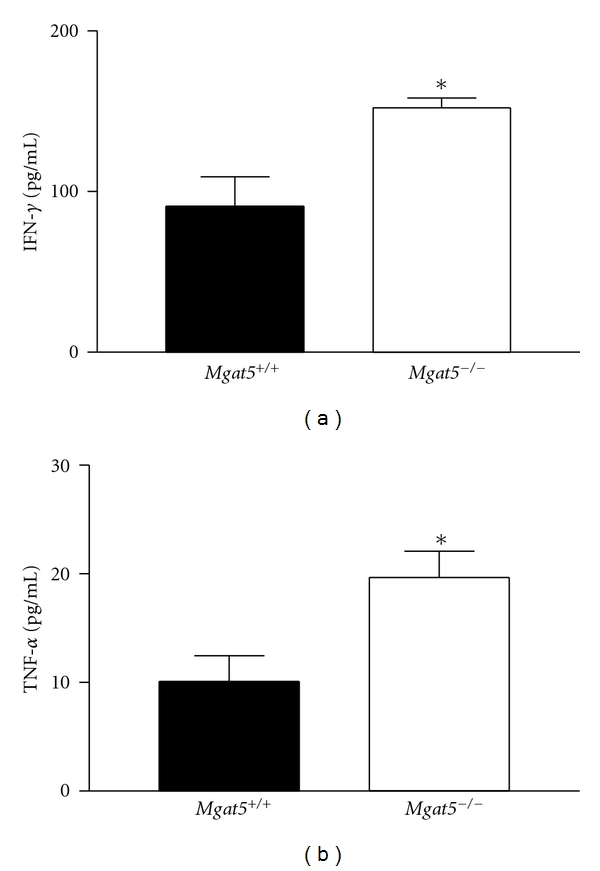
Mice deficient in *Mgat*5 have increased IFN-*γ* and TNF-*α* levels. Splenocytes were harvested from representative mice from both EAE groups and re-stimulated with 50 ug/mL MPB *in vitro*. Supernatants from splenocyte cultures were tested for IFN-*γ* and TNF-*α* levels by a bead-based analyte detection system using flow cytometry. *P ≤ 0.05. *Error bars* indicate the means ± S.E. of duplicate samples.

To further investigate the effects of N-glycan processing deficiency in T-cell-dependent autoimmunity *in vivo*, we utilized an adoptive transfer model of EAE induction. Specifically, we wanted to examine whether N-glycan branching deficiency in self-reactive T cells is sufficient to enhance autoimmune demyelination. EAE may be induced by adoptive transfer of myelin antigen-specific T cells into naïve mice, leading to inflammatory demyelination of axons and progressive motor weakness. Splenocytes from myelin basic protein- (MBP-) immunized *Mgat*5^−/−^ PL/J mice were restimulated *in vitro* with MBP, then purified T cells were transferred into *Mgat*5^+/+^ or *Mgat*5^−/−^ PL/J mice for induction of EAE. Although the same myelin-reactive T cells were injected, the recipient mice deficient in *Mgat*5 displayed dramatically increased incidence and severity of EAE compared with wild-type mice (Figures 2(a) and 2(b)). At the peak of disease, less than 15% of the wild-type mice had disease, whereas greater than 60% of the *Mgat*5^−/−^  mice displayed EAE (Figure 2(a)). Furthermore, the mean highest clinical score and disease duration were significantly increased in the *Mgat*5^−/−^ mice (Figures 2(c) and 2(d)) (Table 1). The *Mgat*5^−/−^ mice also appeared to have slightly greater weight loss, but this was not significantly different (Figure 2(e)). Splenocyte cultures from *Mgat*5^−/−^ mice displayed increased levels of the proinflammatory cytokines IFN-*γ* and TNF-*α* when restimulated with MBP compared with wild-type mice (Figure 3). These results suggest that recipient mice lacking *Mgat*5-modified glycans are more susceptible to EAE autoimmune disease following adoptive transfer of encephalitogenic *Mgat*5^−/−^ T cells, implicating cells of the host's own immune or central nervous system. This raises the possibility that *Mgat*5 deficiency in host, nonantigen-specific T cells and/or non-T cells (i.e., B cells, antigen-presenting cells, and nonimmune cells) contribute to increased susceptibility to inflammatory demyelination. 

## 4. Discussion and Conclusions

During CNS inflammation, antigen-presenting cells (APC) such as invading macrophages and resident microglia can perpetuate the inflammatory milieu by secreting inflammatory factors and presenting myelin epitopes to autoreactive T cells. Endogenous presentation of myelin epitopes by APCs during acute inflammation can initiate epitope spreading and augment the progression of disease [27]. The differences in EAE observed in *Mgat*5^+/+^ versus *Mgat*5^−/−^ mice following adoptive transfer of *Mgat*5^−/−^ MBP-reactive T cells may result from a number of different mechanisms. Hyperactive endogenous T cells in *Mgat*5^−/−^ recipients may respond more vigorously to epitope spreading, thereby enhancing disease. *Mgat*5^−/−^ macrophages have impaired motility and phagocytosis, which may promote inflammatory demyelination and epitope spreading by inhibiting migration away from sites of demyelination and/or clearance of apoptotic cells [28]. Others have suggested that N-glycan branching deficiency in the kidney induced by Golgi *α*-mannosidase-II deficiency may trigger kidney autoimmunity via loss of self-recognition by the innate immune system [29], raising the possibility that reduced branching in oligodendrocytes may similarly activate innate immune cells. Galectin-1 has also been shown to regulate dendritic cell function by increasing tolerogenic signals to T cells and suppressing autoimmune neuroinflammation [30].

Multiple Sclerosis is also characterized by neurodegeneration. Although this may be triggered by inflammatory mediators and/or demyelination, deficiencies in N-glycan branching in neurons/axons may directly contribute to neurodegeneration independent from effects on inflammatory cells. The progressive MS-like disease that spontaneously develops in *Mgat*5^−/−^ PL/J mice displays neuronal loss and axonal damage in both demyelinated areas and otherwise normal appearing white matter, the latter a hallmark of MS [17]. Moreover, neuron-specific deletion of Mgat1, a Golgi enzyme upstream of *Mgat*5 that eliminates all branching in N-glycans, results in apoptosis of adult neurons *in vivo *[31]. This confirms that N-glycan branching is required for neuronal viability in the adult CNS. 

Restoration of neuronal integrity and regeneration of myelinated axons in the damaged CNS is another important mechanism to consider. Neural stem cells residing in the CNS are implicated in regenerating the damaged CNS, and, even within an acute inflammatory brain lesion, spontaneous remyelination occurs [32]. Recently, it has been shown that galectin-1 promotes proliferation of adult neural stem cells in the CNS through its carbohydrate-binding ability, suggesting that N-glycan branching deficiency may also limit regenerative mechanisms in MS and thereby promote progression [33]. Indeed, a polymorphism in *Mgat*5 strongly associates with disease severity in MS [10]. Future investigations are warranted to examine the many potential mechanisms by which deficiency of N-glycan branching by *Mgat*5 and other Golgi enzymes contribute to demyelinating disease initiation and progression. 

##  Conflict of Interests

None of the authors declared conflict of interest.

## Figures and Tables

**Table 1:** Clinical observations of adoptive transfer EAE.

Genotype	*n*	Mean High Score	Incidence		Day of Onset	Mean Duration (Days)
		(Mean ± SEM)	(Day 20)	(Day 40)	(Mean ± SEM)	(Mean ± SEM)
		*P* = 0.009				*P* = 0.014
*Mg* *at*5^+/+^	7	0.3 ± 0.2	0%	0%	9.5 ± 1.5	3.4 ± 2.6
*Mg* *at*5^−/−^	8	1.5 ± 0.3	63%	38%	7.3 ± 1.8	20.8 ± 4.9

Mice were scored daily on a scale of 0–5 with: 0, no disease; 1: loss of tail tone; 2: hindlimb weakness; 3: hindlimb paralysis; 4: forelimb weakness or paralysis and hindlimb paralysis; 5: moribund or dead. *P* values for EAE mean high score and disease duration were determined by the Mann-Whitney test.
